# Diagnostic value of circulating microRNAs for liver cirrhosis: a meta-analysis

**DOI:** 10.18632/oncotarget.23332

**Published:** 2017-12-16

**Authors:** Liwei Guo, Weiyan Li, Liyang Hu, Huanhuan Zhou, Lei Zheng, Lifei Yu, Weifeng Liang

**Affiliations:** ^1^ State Key Laboratory for Diagnosis and Treatment of Infectious Diseases, Collaborative Innovation Center for Diagnosis and Treatment of Infectious Diseases, The First Affiliated Hospital, College of Medicine, Zhejiang University, Hangzhou, China; ^2^ Shengzhou People's Hospital, Shengzhou Branch of the First Affiliated Hospital of Zhejiang University, Shengzhou, China; ^3^ Shanghai Ninth People's Hospital, Shanghai Jiaotong University School of Medicine, Shanghai, China; ^4^ Institute of Cancer Research, Zhejiang Cancer Hospital, Hangzhou, China; ^5^ Department of Infectious Diseases, Hangzhou First People's Hospital, Nanjing Medical University, Hangzhou, China

**Keywords:** liver cirrhosis, circulating, microRNA, diagnosis, meta-analysis

## Abstract

Circulating microRNAs are potential biomarkers for various diseases including liver cirrhosis. We designed a meta-analysis to evaluate the diagnostic value of circulating microRNAs for liver cirrhosis patients. Eligible studies were identified by searching PubMed, Embase, and the Cochrane Library up to July 1, 2017. The diagnostic sensitivity, specificity, positive likelihood ratio (PLR), negative likelihood ratio (NLR), diagnostic odds ratio (DOR), and area under the receiver operating characteristic (AUROC) curve were analyzed using a random or fixed effects models based on the between-study heterogeneities. Thirteen studies from 7 articles with 627 patients and 418 healthy controls were included in this meta-analysis. All studies had high quality assessment scores. The pooled sensitivity, specificity, PLR, NLR, DOR and AUROC were 0.83 (95% CI: 0.80–0.86), 0.89 (95% CI: 0.86–0.92), 6.41 (95% CI: 3.93–10.44), 0.22 (95% CI: 0.14–0.33), 35.18 (95% CI: 15.90–77.81) and 0.93 (95% CI: 0.91–0.95), respectively. In conclusion, circulating microRNAs may serve as potential noninvasive biomarkers of liver cirrhosis.

## INTRODUCTION

MicroRNAs (miRNAs) are small, endogenous, noncoding, 18–24 nucleotide RNAs that can regulate gene expression by base pairing with the 3′-untranslated regions (UTRs) of target messenger RNAs [1, 2]. MiRNAs have been reported to contribute the regulation of a diverse range of genetic processes including development, apoptosis and differentiation. The expressions of circulating miRNAs are stable, reproducible and consistent among individuals of the same species [3]. MiRNAs are receiving increasing attention for their potential as diagnostic and therapeutic targets [4–6]. Many studies have reported that serum miRNAs have been identified as fingerprints for numerous diseases and cancers [7, 8].

Liver cirrhosis is a pathological condition of liver that results from sustained wound healing in response to various causes of chronic liver injury, including chronic hepatitis B (CHB), chronic hepatitis C (CHC), autoimmune hepatitis and alcoholic hepatitis [9]. The exact prevalence of cirrhosis worldwide is unknown. More than one million deaths worldwide were attributed to cirrhosis in 2010, although these figures are probably heavily under-reported. The total worldwide prevalence of cirrhosis has been estimated to be approximately 1% with significant regional variation due to the presence of viral hepatitis, metabolic syndrome and alcohol consumption [10]. Cirrhosis results in 170,000 deaths per year in Europe and 33,539 deaths per year in the USA. The main causes in these countries are infection with hepatitis C virus, alcohol misuse and non-alcoholic liver disease. The numbers in Europe and the USA are even higher than those in most Asian and African countries where chronic viral hepatitis B and C are common [11]. The prevalence of cirrhosis is difficult to assess and probably higher than reported because the initial stages are asymptomatic, so the disorder is undiagnosed.

Liver biopsy is considered as the gold standard for evaluating fibrosis [12]. However, it is limited by the need for hospital admission and sedation in children. It is not widely accepted by patients due to its limitations, especially the serious risks that include bleeding. The accuracy of liver histology assessment has been challenged because of sampling errors because, for example, the liver specimens are small or fragmented [13]. Advanced imaging technologies, including magnetic resonance imaging (MRI), computed tomography (CT), and transient elastography (TE) may also be used to detect liver fibrosis, but the use of these methods in countries such as China is limited by their high costs and lack of availability in many medical institutions. The diagnostic accuracy of CT, MRI and TE may be influenced by many factors, including obesity, ascites, acute inflammation, liver congestion, and elevated portal vein pressure [14, 15]. Many non-invasive markers for assessing liver cirrhosis are applied in clinical practice but are not sufficiently accurate because of low sensitivities and specificities [16]. Noninvasive fibrosis indices, such as the APRI and FIB-4, are associated with lower costs, do not require particular expertise in their interpretation, and can be performed in an outpatient setting. However, these indices have limited use in distinguishing patients who have chronic hepatitis from those who have developed cirrhosis [17].

In the past, an overwhelming amount of data supporting a role for miRNAs in the development and progression of chronic liver diseases into liver cirrhosis and finally hepatocellular carcinoma (HCC) has been presented [18, 19]. Various miRNAs have been reported to be correlated with liver cirrhosis and could be used as novel non-invasive biomarkers, including miRNA122, miRNA181b, miRNA29, etc. [20–23]. Therefore, we conducted a systematic and comprehensive meta-analysis of all eligible studies to explore the overall diagnostic values of serum miRNAs as promising biomarkers of liver cirrhosis.

## RESULTS

### Search results and characteristics of the eligible studies

**Figure 1:**
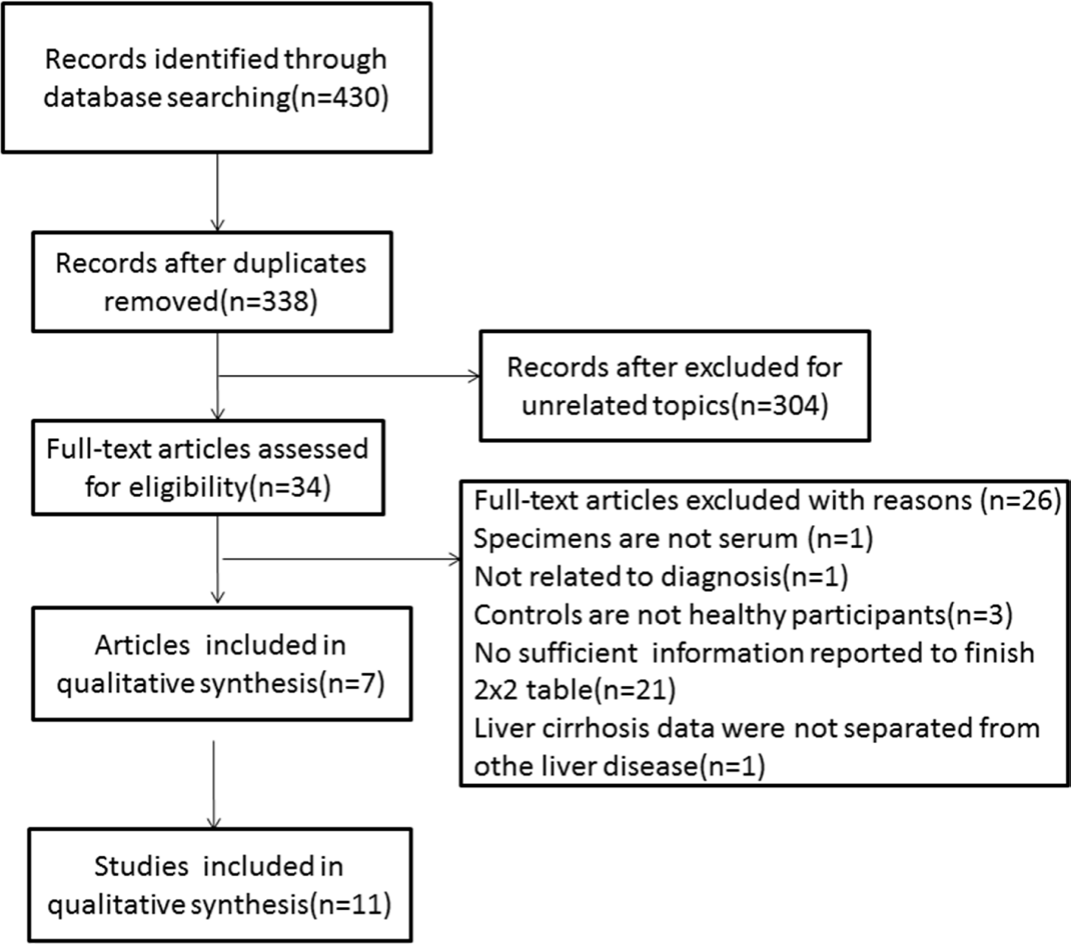
Flow diagram of study inclusion and exclusion for meta-analysis

**Figure 2:**
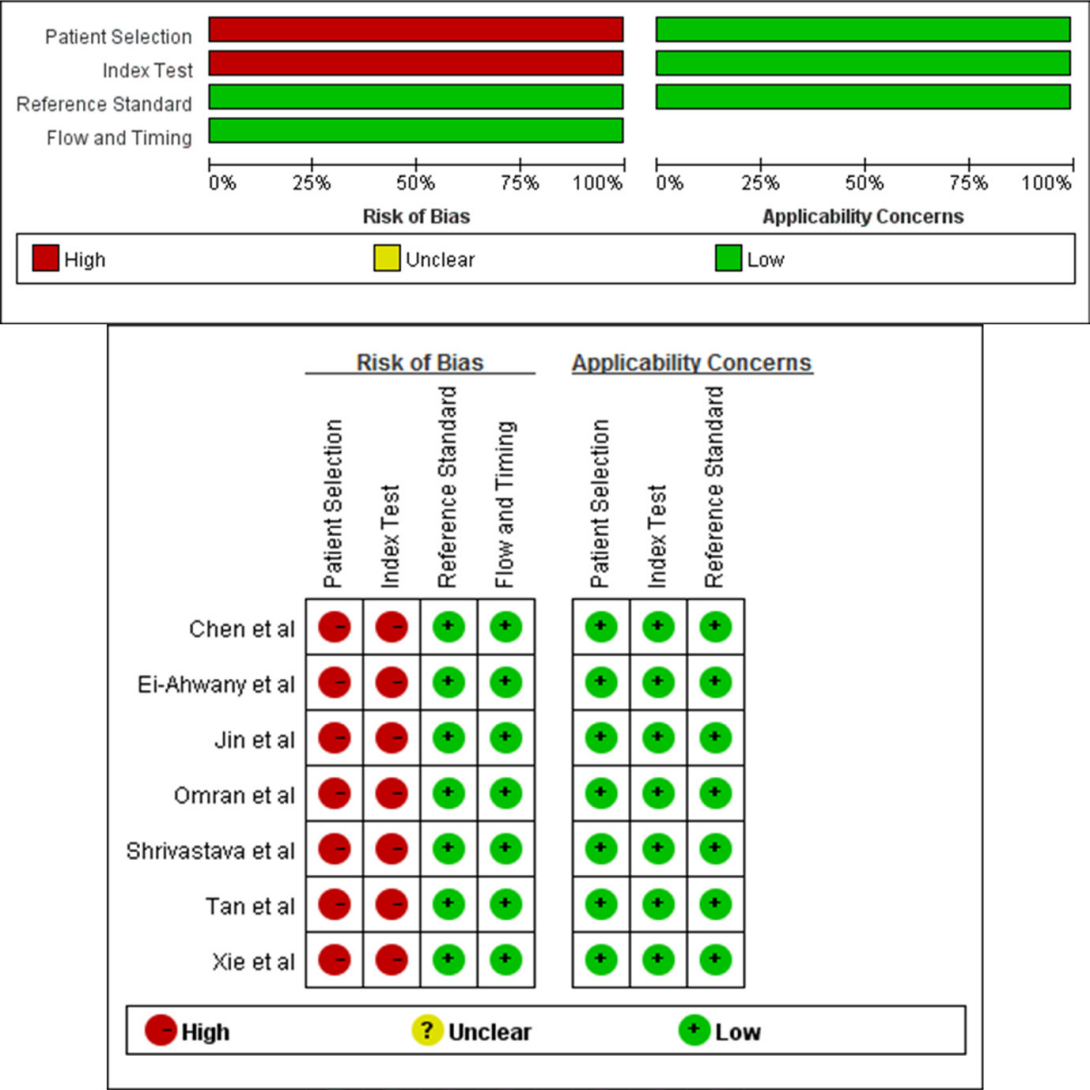
Details of QUADAS-2 quality assessment of each included study (QUADAS-2 tool)

We searched 430 records of which 92 records were duplicates. After a primary screening of the titles and abstracts, 304 records were excluded. Further articles were excluded after review. Seven eligible articles with 627 patients were included in our meta-analysis [33–39] (Figure 1). The characteristics of all 8 of the included publications are provided in Table 1. The included publications were published between 2013 and 2016. All 1045 described subjects were included. The results of the QUADAS-2 study quality assessments are presented in Figure 2. The majority of all articles included in the current meta-analysis satisfied most of the items in the QUADAS-2, which suggest that the overall quality of included studies was moderate-high.

**Table 1:** Characteristics of eligible studies

Author	Year	Country	MicRNA	Number	Liver disease	Fibrosis stage	TP	FP	FN	TN	Sensitivity	Specificity
Ei-Ahwany *et al*.	2016	Egypt	miR-138	66	CHC	early fibrosis	59	11	7	29	0.893	0.714
Ei-Ahwany *et al*.	2016	Egypt	miR-138	65	CHC	late fibrosis	58	3	7	37	0.893	0.930
Ei-Ahwany *et al*.	2016	Egypt	miR-143	65	CHC	late fibrosis	49	5	16	35	0.75	0.884
Chen *et al*.	2013	China	miR-106b+181b	13	CHB	cirrhosis	8	0	5	6	0.615	0.935
Chen *et al*.	2013	China	miR-106b+181b	47	non-CHB	cirrhosis	37	3	10	35	0.787	0.932
Omran *et al*.	2015	Egypt	miR-20a	40	CHC	fibrosis	40	0	0	20	1	1
Shrivastava *et al*.	2013	USA	miR-122	44	CHC	fibrosis	27	4	17	18	0.614	0.818
Shrivastava *et al*.	2013	USA	miR-92a	44	CHC	fibrosis	31	5	13	17	0.705	0.773
Tan *et al*.	2014	China	miRNA panel	82	PBC	cirrhosis	66	7	16	53	0.805	0.883
Jin *et al*.	2015	China	miRNA panel	100	CHB	cirrhosis	95	2	5	98	0.95	0.98
Xie *et al*.	2014	China	miR-101	61	CHB	cirrhosis	49	6	12	24	0.803	0.8

Abbreviations: PBC: Primary Biliary Cirrhosis, CHC: Chronic hepatitis C, CHB: Chronic hepatitis B, TP: true positive, FP, false positive, TN: true negative, FN, false negative.

### Diagnostic accuracy of the serum miRNAs in the detection of liver cirrhosis

**Figure 3:**
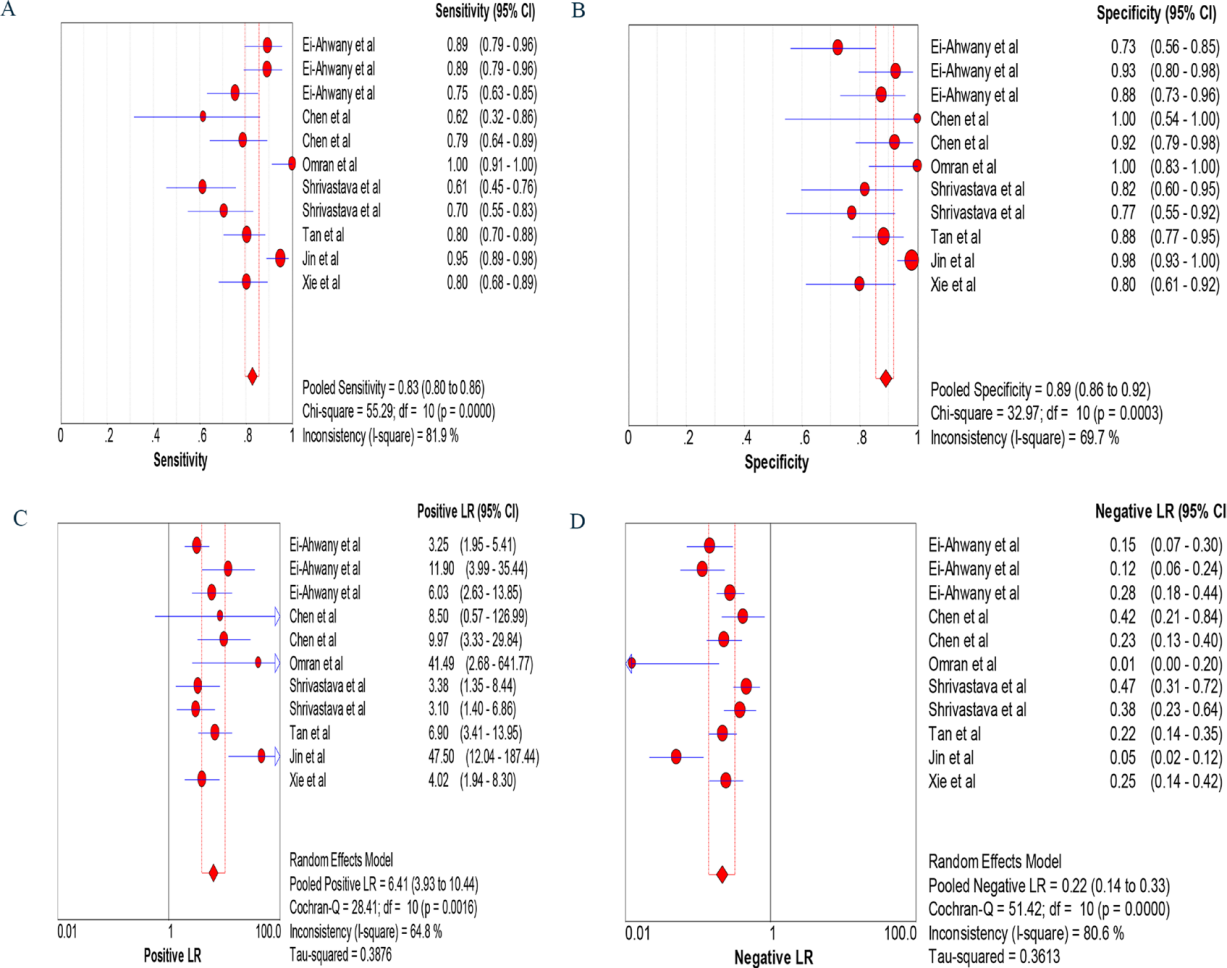
Forest plots of sensitivity (**A**), specificity (**B**), positive likelihood ratio specificity (**C**) and negative likelihood ratio specificity (**D**). The width of the horizontal line represents the 95% CI of each study; square proportional means the weight of every study. The weight is evaluated by the sample size and is presented as percent of total. The diamond represents pooled sensitivity, specificity, positive likelihood ratio specificity, negative likelihood ratio specificity and 95% CI.

**Figure 4:**
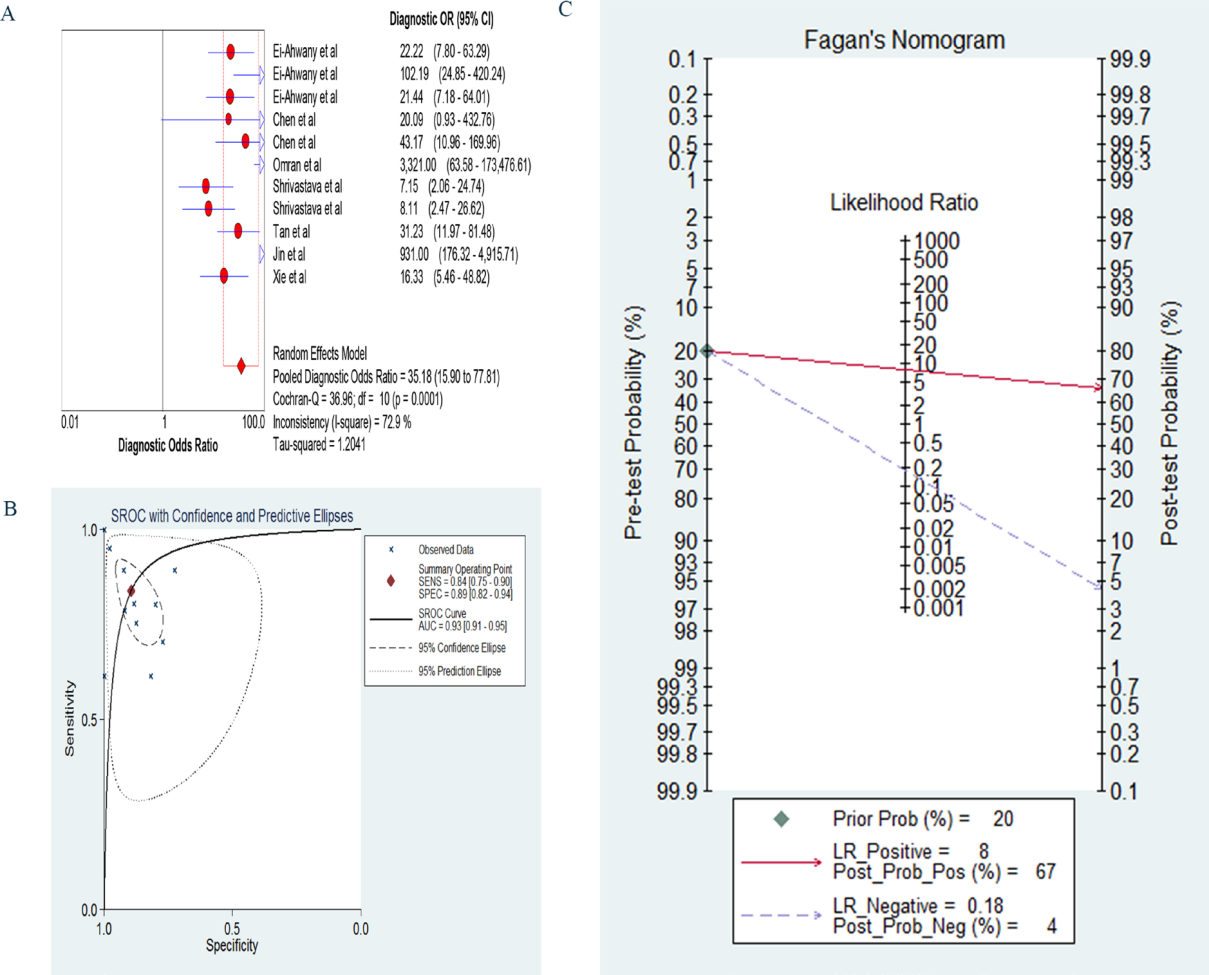
(**A**) The forest of diagnostic odds ratio specificity; (**B**) The pooled receiver operating characteristic curve: each X mark represents a study and AUC is the area under the curve; (**C**) Fagan's Nomogram for calculation of post-test probabilities.

The sensitivity was calculated with the random effects model, and the pooled sensitivity was 0.83 (95% confidence interval [CI]: 0.80–0.86; Figure 3A). The pooled diagnostic specificity was 0.89 (95% CI: 0.86–0.92) based on the random effects model (Figure 3B). The pooled PLR was 6.41 (95% CI: 3.93–10.44) based on the random effects model (Figure 3C). The pooled NLR was 0.22 (95% CI: 0.14–0.33) based on the random effects model (Figure 3D). The DOR was pooled in a random effects model, and the pooled DOR was 35.18 (95% CI: 15.90–77.81; Figure 4A). The area under the ROC curve was 0.93 (95% CI: 0.91–0.95) (Figure 4B). To evaluate the clinical utility of the index test, a Fagan's nomogram was created to predict the increasing inerrability of a positive diagnosis using the value of the test, and it was used to estimate the post-test probabilities (Figure 4C). The PLR was 8, the NLR was 0.18, and the post-test probabilities were 67 and 4, respectively. These data demonstrate that circulating miRNAs can be assayed with high diagnostic accuracy and specificity.

### Statistical heterogeneity of the included studies

Differences in cut-off values lead to the threshold effect. When there is a threshold effect, an inverse correlation is present between the sensitivity and specificity. The Spearman correlation coefficient was −0.318, and the *p* value was 0.340 (*P* > 0.05), which indicated that there was no significant threshold effect.

### Publication bias and sensitivity analysis

**Figure 5:**
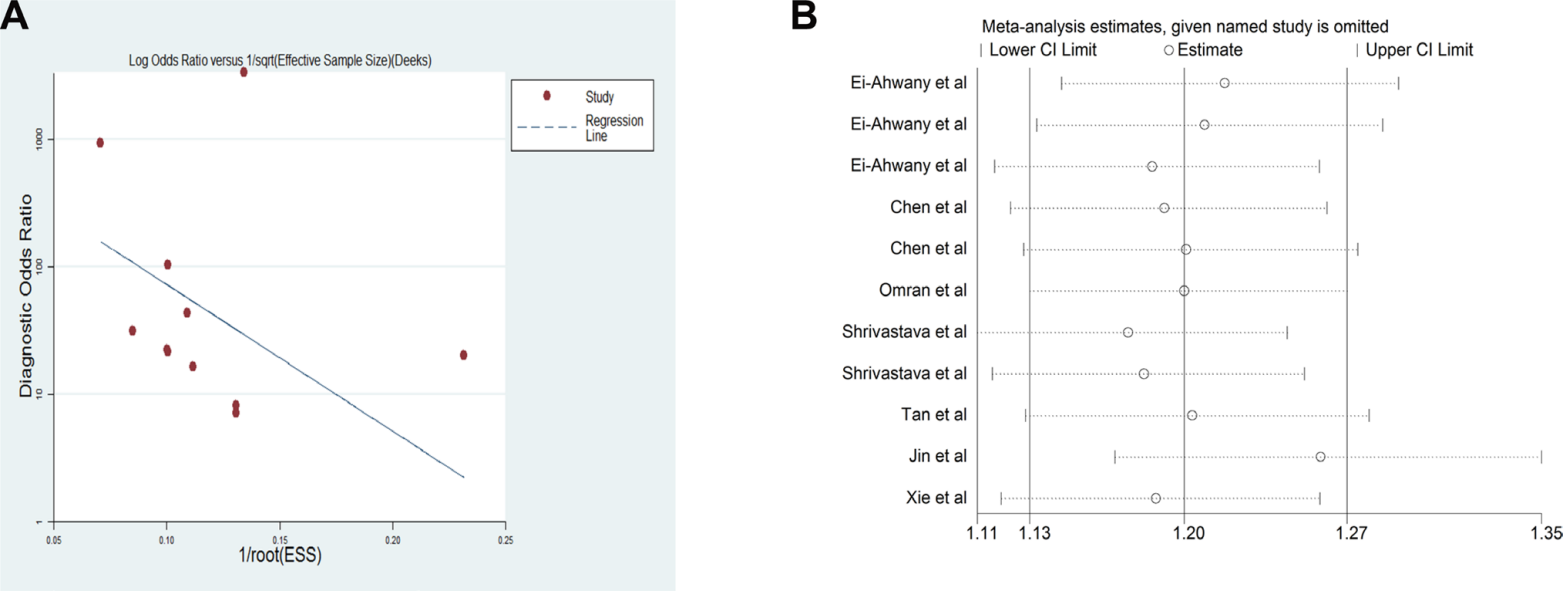
(**A**) Deek's funnel plot indicates no significant publication bias (*p* = 0.239 > 0.05); (**B**) Sensitivity analysis plot of meta-analysis. Every row represents an included study. The width of the horizontal line represents the 95% CI for each study. The vertical bar on both sides represents the lowest and highest values of 95% CI.

A Deek's funnel plot was constructed, and the asymmetry test was performed to explore any potential publication bias in this meta-analysis. No significant publication bias was discovered (*P* = 0.239 > 0.05; Figure 5A). The sensitivity analysis is presented in Figure 5B; this analysis was accomplished by excluding studies one by one. The data were stable and not significantly different.

### Sub-group analysis

The sub-group analysis is presented in Table 2. The sample size (>60 or ≤60), miRNA type (single or combined) and liver disease (CHC or others) are displayed. The data suggested that the combined miRNAs exhibited greater diagnostic accuracy than the single miRNAs. The results were less accurate for the CHC cirrhosis patients than those with liver cirrhosis resulting from other diseases.

**Table 2:** Detail information of subgroup analysis

Subgroup	Patients	Sensitivity	Specificity	PLR	NLR	DOR	AUC
Total	685	0.83 (0.80–0.86)	0.89 (0.86–0.92)	6.41 (3.93–10.44)	0.22 (0.14–0.33)	35.18 (15.90–77.81)	0.93 (0.91–0.95)
Sample size							
>60	503	0.856 (0.820–0.888)	0.890 (0.850–0.923)	7.105 (3.640–13.868)	0.166 (0.103–0.266)	46.702 (17.539–124.35)	0.93 (0.91–0.95)
≤60	182	0.761 (0.693–0.802)	0.889 (0.814–0.941)	5.460 (2.500–11.924)	0.323 (0.184–0.567)	22.865 (5.789–90.313)	0.94 (0.91–0.96)
MiRNA type							
single	385	0.813 (0.770–0.851)	0.841 (0.785–0.887)	4.512 (2.944–6.916)	0.234 (0.144–0.382)	22.307 (9.778–50.889)	0.92 (0.89–0.94)
combined	300	0.851 (0.880–0.894)	0.941 (0.900–0.969)	12.417 (4.756–32.420)	0.189 (0.086–0.417)	75.885 (14.895–386.60)	0.95 (0.93–0.97)
Liver disease							
CHC	241	0.815 (0.768–0.856)	0.848 (0.788–0.896)	4.790 (2.797–8.203)	0.225 (0.123–0.411)	24.874 (9.058–68.286)	0.93 (0.90–0.95)
Other	444	0.842 (0.796–0.881)	0.923 (0.881–0.951)	9.222 (3.974–21.400)	0.202 (0.112–0.366)	52.854 (14.231–196.29)	0.94 (0.90–0.95)

## DISCUSSION

Currently, circulating miRNAs are attracting increasing attention for their high stability and great potential as biomarkers for various diseases and cancers [40–42]. Liver cirrhosis is the advanced stage of chronic liver diseases. Liver biopsy is not widely accepted by patients due to its limitations. Many non-invasion biomarkers have been used in the detection of liver cirrhosis, including the APRI and FIB-4. However, the diagnostic accuracies are inconsistent [43–45]. Previous studies have reported miRNAs as biomarkers of liver cirrhosis [46, 47]. This study is the first meta-analysis to assess the diagnostic value of circulating miRNAs in identifying liver cirrhosis. This meta-analysis was conducted with multiple searching strategies performed by independent reviewers according the inclusion and exclusion criteria.

Eleven studies from 7 articles with 1045 subjects (627 patients and 418 healthy controls) were included in this meta-analysis. The 11 included studies exhibited moderate or high sensitivity and specificity, as the sensitivities ranged from 0.614 to 1, and the specificities ranged from 0.714 to 1. The results of our meta-analysis indicated that circulating miRNAs presented satisfactory pooled sensitivity and specificity. The pooled sensitivity was 0.83 (95% CI, 0.80–0.86), and the pooled specificity was 0.89 (95% CI, 0.86–0.92); thus, circulating miRNAs represent a promising diagnostic marker for liver cirrhosis. We also used the SROC curve and the corresponding AUC to estimate the overall diagnostic performance in the meta-analysis. The evaluation criteria can be divided into 3 levels of accuracy: low (AUC: 0.5–0.7), moderate (AUC: 0.7–0.9), and high (AUC: 0.9–1) [48]. In this meta-analysis, the AUC value for liver cirrhosis was 0.93, which indicated a high level of overall accuracy.

To further evaluate the diagnostic accuracy, we analyzed the DOR, PLR and NLR. The DOR represents the discrimination ability of a diagnostic test and ranges from 0 to infinity; the greater DORs indicate greater discriminative abilities. In this meta-analysis, the DOR value was 35.18, which indicates that the overall accuracy of the circulating miRNAs for the diagnosis of liver cirrhosis was credible. The LRs indicate the amount by which the odds of disease increase or decrease with a positive or negative test result [49]. The probability of a true positive and the value of the PLR exhibit a direct ratio when the test is positive. Higher NLR values indicate higher probabilities of false-negatives when the test is negative. When the PLR>10 or the NLR<0.1, the likelihood of diagnosis or exclusion of a disease increases significantly [49]. Nevertheless, the PLR of 6.41 (95% CI, 3.93–10.44) and the NLR of 0.22 (95% CI: 0.14–0.33) indicated that patients with liver cirrhosis have a ∼6.41-fold higher chance of testing positive based on the circulating miRNAs than the controls, and 22% of individuals with liver cirrhosis will have a negative result.

LRs and post-test probabilities are correlations for clinicians because they provide information about the likelihood of a patient with a positive or negative test actually exhibiting liver cirrhosis [25, 49]. From the Fagan's nomogram, we found that, when a pre-test probability of 20% was specified, the post-test probability of positivity increased to 67% with a positive likelihood ratio of 8, and the post-test probability of negativity decreased to 4% with a negative likelihood ratio was 0.18. These outcomes suggest a stable value of circulating miRNAs in the diagnosis of liver cirrhosis.

The threshold effect is one of the causes of heterogeneity in diagnostic accuracy studies. We did not find obvious heterogeneity as a result of heterogeneity in our meta-analysis. Therefore, we performed a meta-regression to examine the effects of sample size, liver disease type and whether single or combined miRNAs were utilized. The results revealed that sample size was a potential source of heterogeneity in this meta-analysis. The sub-group analysis revealed that the diagnostic sensitivity, specificity, PLR, DOR and AUC for liver cirrhosis in the subgroup of combined miRNAs were greater than the corresponding values in the single miRNA subgroup. These findings indicate that combined miRNAs have higher diagnostic value than single miRNAs. Additionally, the sensitivity analysis and the Deek's funnel plot revealed that there were no outliers or a significant publication bias (*p* = 0.239).

This meta-analysis has several limitations. First, despite extensive literature search were performed, the number of included studies and sample sizes were small, which may restrict our ability to evaluate the accuracy of circulating miRNAs in detecting liver cirrhosis. Therefore, more large-scale, well-designed and multi-center clinical researches should be performed before the application of circulating miRNAs for the diagnosis of patients with liver cirrhosis. Second, there was significant heterogeneity in this meta-analysis; the Spearman correlation coefficient data indicated that the heterogeneity was not due to a threshold effect. Thus, the heterogeneity may have primarily been due to the small sample sizes. We attempted to establish a subgroup analysis for the disease stages, but because one study did not discriminate fibrosis and cirrhosis, this was difficult. Future studies should be designed to evaluate the heterogeneity. Finally, the demographics of the studies were limited to three countries. We have reviewed records from other countries, but these studies did not meet the inclusion criteria. Thus, it remains unknown whether these findings may be applicable to other parts of the world.

In conclusion, this meta-analysis demonstrated that circulating miRNAs can serve as potential biomarkers of liver cirrhosis. However, further large-scale studies are needed to confirm our analyses.

## MATERIALS AND METHODS

### Searching strategy and study selection

We reviewed publications in PubMed, EMBASE and the Cochrane Library up to July 1, 2017. We used the following search terms: (‘serum’ or ‘plasma’ or ‘circulating’ or ‘blood’) and (‘microRNA’ or ‘miRNA’) and (‘liver fibrosis’ or ‘liver cirrhosis’) and (‘biomarker’ or ‘diagnosis’). Only studies published in English were included.

### Inclusion and exclusion criteria

The inclusion criteria for the primary studies were as follows: (1) the study was a diagnostic study using serum miRNA; (2) the subjects included liver cirrhosis patients and healthy controls; and (3) sufficient information was available to construct 2 × 2 tables that consisted of the true positives (TPs), false positives (FPs), true negatives (TNs) and false negatives (FNs). Articles were excluded if the miRNAs were not detected using serum samples, if there was insufficient information reported to create a 2 × 2 table, and if the control groups were not healthy participants. The studies included in our meta-analysis were independently assessed by two investigators. All of the selected studies were managed using EndNote X7.

### Data extraction and quality assessment

The first author's name, year of publication, country, number of patients, miRNA type, liver disease, fibrosis stage and detection methods were collected from the eligible studies. Then, 2 × 2 tables that displayed the TP, TN, FP, and FN were created. The Quality Assessment of Diagnostic Accuracy Studies-2 (QUADAS-2) was used to evaluate the diagnostic accuracy qualities using the patient selection, index test, reference standard, and flow and timing [24].

### Statistical analysis

For the diagnostic meta-analysis, the accuracy indicators included the pooled sensitivity (SEN), pooled specificity (SPE), positive likelihood ratio (PLR), negative likelihood ratio (NLR), diagnostic odds ratio (DOR), and their 95% confidence intervals (CIs) were calculated using the random-effects model [25, 26]. The PLR was based on the odds of positive test results for liver cirrhosis patients, and the NLR reflected the odds of positive results for those without cirrhosis. The DOR was the outcome of the combination of the PLR and NLR (DOR = PLR/NLR) [27]. Simultaneously, the summary receiver operator characteristic (SROC) curve was created, and the area under the SROC curve (AUC) was calculated. The analysis of the diagnostic accuracy was pursuant to a SROC curve and the AUC of the SROC [28, 29]. The heterogeneity was measured with the *I*^2^ and *Q*-test, and a *P* < 0.05 and an *I*^2^ > 50% indicated the existence of significant heterogeneity among studies. If heterogeneity was detected, the random effects model was employed; otherwise, the fixed effects model was used. Meta-regression was used to detect the potential heterogeneity among the included studies [30]. Additionally, the Spearman correlation coefficient was used to verify if the heterogeneity in the meta-analysis could be explained by a threshold effect. A threshold effect was defined as a positive correlation (*P* < 0.05) [31]. Publication bias was investigated using Deek's funnel plot [32]. Sensitivity analysis was accomplished by excluding the studies one by one.

The data analyses were performed using the Meta-Disc statistical software version 1.4 (XI Cochrane Colloquium, Barcelona, Spain) and STATA software (version 12.0, STATA Corp, MIDAS module). Quality assessment was managed by Review Manager 5.3 (Cochrane Collaboration, Copenhagen, Denmark). A *p* value < 0.05 was considered statistically significant.
